# Activation of Stimulation of Interferon Genes (STING) Signal and Cancer Immunotherapy

**DOI:** 10.3390/molecules27144638

**Published:** 2022-07-20

**Authors:** Kewang Luo, Ning Li, Wei Ye, Hanchao Gao, Xinle Luo, Baohui Cheng

**Affiliations:** 1 Department of Rehabilitation Medicine, People’s Hospital of Longhua, The Affiliated Hospital of Southern Medical University, Shenzhen 518109, China; kewangluo@126.com (K.L.); luoxinlesz@163.com (X.L.); 2 Shenzhen Middle School, Shenzhen 518001, China; ninglee5933@outlook.com; 3 School of Pharmacy, Southern Medical University, Guangzhou 510505, China; yeyewei2022@163.com; 4 Department of Nephrology, Shenzhen Longhua District Central Hospital, Affiliated Central Hospital of Shenzhen Longhua District, Guangdong Medical University, Shenzhen 518110, China; hcgao@foxmail.com; 5 Longgang E.N.T Hospital & Shenzhen Key Laboratory of E.N.T, Department of Otolaryngology, Institute of E.N.T Shenzhen, Shenzhen 518172, China

**Keywords:** cGAS-STING pathway, cancer immunotherapy, STING agonists

## Abstract

Stimulator of interferon gene (STING), an intracellular receptor in the endoplasmic reticulum, could induce the production of cytokines such as type I interferon (IFN) by activating the cGAS-STING signal pathway. In recent years, activation of STING has shown great potential to enhance anti-tumor immunity and reshape the tumor microenvironment, which is expected to be used in tumor immunotherapy. A number of STING agonists have demonstrated promising biological activity and showed excellent synergistic anti-tumor effects in combination with other cancer therapies in preclinical studies and some clinical trials. The combination of STING agonists and ICI also showed a potent effect in improving anti-tumor immunity. In this review, we introduce the cGAS-STING signaling pathway and its effect in tumor immunity and discuss the recent strategies of activation of the STING signaling pathway and its research progress in tumor immunotherapy.

## 1. Introduction

It is widely accepted that the immune system has critical roles in the host defense in the recognition and elimination of malignancies. Innate immunity, referring to the immune ability of an individual at birth, is the first line of the host defense against infection of various pathogenic microorganisms. Innate immunity recognizes non-self-components through pattern recognition receptors to resist pathogen invasion and tissue damage [1]. After the activation of pattern recognition receptors, the innate immune response and subsequent adaptive required immune response are induced. During the process, DNA is an effective immune-stimulating molecule, which is widely used as a vaccine adjuvant to trigger immunity. The stimulator of interferon gene (STING) pathway is a critical cytoplasmic DNA-sensing process, which induces innate immunity against microorganism and bacteria [2].

STING, an intracellular recognition receptor, has important roles in modulating the transcription of many guard genes by activating the cGAS-STING signal pathway [3]. STING is located on the membrane of the endoplasmic reticulum (ER), and contains 379 amino acids, including 4 transmembrane helices and a spherical C-terminal domain (CTD) extending into the cytoplasm [4]. In 2008, STING was revealed to be an important ingredient in DNA-mediated innate immunity in response to the invasion of bacteria, DNA viruses, etc. [3,5,6]. Further investigation by Chen et al. demonstrated that cGAS is the cytosolic sensor that activates innate immunity by triggering type I interferon (IFN) expression [7,8]. Cytoplasmic DNA stimulates the cascade of cGAS-STING signaling. This signaling is involved in the defense against microorganisms and was determined to be effective in improving anti-tumor immunity and other autoimmune diseases.

In this review, we discuss the cGAS-STING signaling pathway and the efficacy in cancer immunity, emphasizing the strategies for activating the STING signaling pathway in cancer treatment and summarizing the clinical applications and research progress of the cGAS-STING signaling pathway in caner immunotherapy in recent years.

## 2. cGAS-STING Signaling Pathway and Cancer Immunotherapy

The cGAS-STING signaling pathway is an important cytoplasmic DNA-sensing pathway in vivo, which takes part in regulating pathogen infection, cancer immune response, and autoimmune diseases by triggering the production of type I IFN. In 2008, Ishikawa et al. [3,6] and Zhong et al. [5] both discovered a new transmembrane protein STING, and the second messenger cyclic guanosine diphosphate (c-di-GMP) in bacteria, which could activate the STING-dependent immune system, was also reported later. In 2013, Chen et al. identified that cyclic guanosine monophosphate adenosine monophosphate synthase (cGAS) is a direct cytoplasmic DNA transducer. cGAS activates interferon regulatory factor 3 (IRF3) by binding to DNA in the cytoplasm, and then activates the production of type I IFN in the form of STING dependence, and triggers innate immunity [7]. It was demonstrated that the STING signal pathway can be induced by cytoplasmic DNA accumulation, such as pathogen DNA and damaged double- or single-stranded DNA (dsDNA or ssDNA) [9,10]. cGAS is a DNA recognition receptor. When cGAS accumulates with DNA under the circumstances of the cytoplasm, cGAS is activated and binds to dsDNA, and then catalyzes adenosine 5′-triphosphate (ATP) and guanosine 5′-triphosphate (GTP) to form cyclic guanosine monophosphate–adenosine monophosphate (cGAMP) [11].

**Figure 1:**
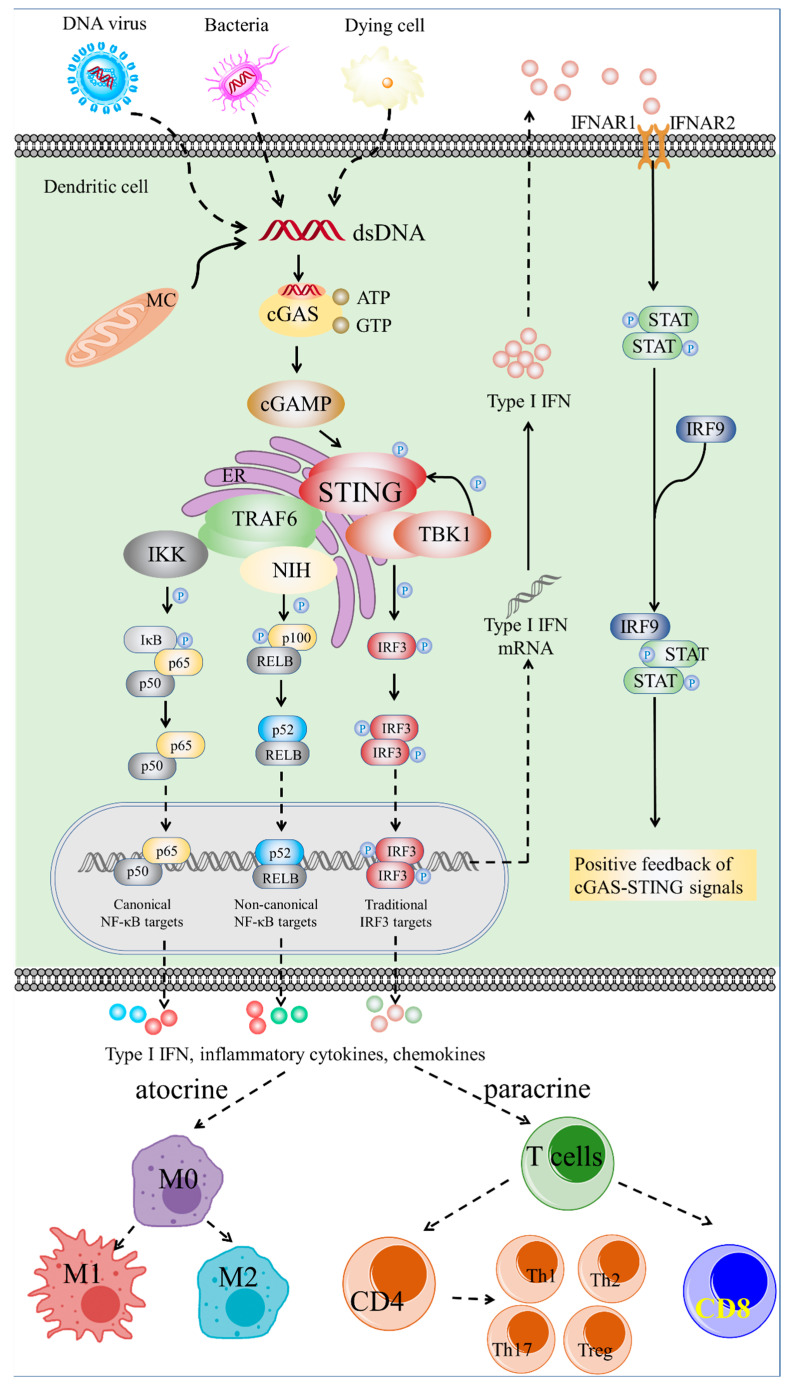
The cGAS-STING pathway. The cytosolic DNA sensor cGAS interacts with exogenous DNA from dying cells, virus, and bacteria, which promotes a conformational change in cGAS to catalyze the formation of cGAMP. The cGAS activation and cGAMP synthase activate protein STING. TBK1 and STING co-phosphate IRF3. Dimerized IRF3 imports to the nucleus to target corresponding genes. IRF3 regulates the expression of IFNB1 in the nucleus. IFNB1 translation in the cytoplasm results in the production of type I IFN, secreting out of cells. Type I IFN stimulates tyrosine kinase-associated receptor and IFNAR1/IFNAR2 heterodimers, which phosphorylates STAT1/STAT2. IRF9 together with phosphorylated dimer STAT/STAT, as a transcriptional factor, transactivates cGAS, developing the positive feedback of cGAS-STING signals. Similarly, mitogen-activated protein kinase 14 and IKK are recruited by activated STING. NIK phosphorylates nuclear factor kappa B subunit 2 (NFKB2/p100) combined with RELB. After degradation of phosphorylated p100 to p52, p52 and REBL form a heterodimer to elicit non-canonical NF-κB signals. For the canonical NF-κB signals, kinase IKK phosphorylates NF-κB inhibitor alpha to recognize proteasomal degradation. Thus, the heterodimer p65/p50 is separated from the IκB/p65/p50 complex to the nucleus, eliciting canonical NF-κB signals. Activation of the cGAS-STING signal pathway induces a series of immune cascades to produce diverse products, including type I IFN, inflammatory cytokines, and chemokines. In an autocrine way, it could promote the maturation, activation, and polarization of macrophages. In a paracrine way, the different cytokines produced by APCs could recruit T lymphocytes and promote their proliferation and differentiation. All the above immune responses participate in the pathogenesis and progression of various diseases. Abbreviations: cGAS, cyclic GMP-AMP synthase; cGAMP, 2′,3′-cyclic GMP-AMP; ER, endoplasmic reticulum; IFN, interferon; IFNAR1, Interferon Alpha And Beta Receptor Subunit 1; IFNAR2, Interferon Alpha And Beta Receptor Subunit 2; IKK, IkB kinase; IFNB1, Interferon Beta 1; IRF, interferon regulatory factor; JAK-STAT, the Janus kinase-signal transducer and activator of transcription; NF-κB, nuclear factor kappa-light-chain-enhancer of activated B cells; STING, stimulator of interferon genes; NIK, NF-κB-inducing kinase; RELB, RELB proto-oncogene; TBK1, TANK-binding kinase 1.

As a secondary messenger, cGAMP combines with STING on the film of ER. Then, STING is rapidly dimerized and activated, and transfers from ER to the Golgi apparatus. Then, kinases such as TANK-binding kinase 1 (TBK1) and IKB kinases (IKKs) in the Golgi apparatus are recruited, and these kinases phosphorylate IRF3, STING, and IκBα (the inhibitor of NF-κB). As a signaling adaptor, the phosphorylated STING collects IRF3, which forms a homodimer and transfers to the nucleus, activating the transcription and translation of type I IFN and other cytokines [12,13]. On the other hand, the phosphorylation of IκBα leads to the transfer of NF-κB to the nucleus, where it triggers the transcription and translation of proinflammatory cytokines such as interleukin-6 (IL-6), tumor necrosis factor (TNF), and type I IFN [14], having an immunomodulatory effect. Type I IFNs combine with heterodimer interferon receptors (IFNAR1 and IFNAR2), and lead to the gathering of Janus family kinase1 (Jak1) and tyrosine kinase 2 (Tyk2), which, in turn, phosphorylate IFNAR1 and IFNAR2. The activated IFNAR1 and IFNAR2 induce the phosphorylation of the STAT family, which, along with IRF9, metastasize to the nucleus and increase the transcription of target genes [15], resulting in the activation of innate and adaptive immunity (Figure 1).

The expression of STING is inhibited in most tumors, which is tumor type specific. It has been demonstrated that STING has low expression in pancreatic cancer [16], and STING is gradually defected in colon adenocarcinoma from stage II to the advanced stage, as assessed by immunohistochemical staining and RNA analysis [17]. In malignant melanoma, the inhibition of STING is also related to the tumor stage [18]. According to the current research, the expression of STING is usually inhibited in most cancer types, rather than being upregulated, especially in advanced tumors [19]. When the cGAS-STING pathway in tumor cells is activated, cytokines such as IL-6 and type I IFN are induced, leading to tumor cell apoptosis or death. Moreover, the released dsDNA and other tumor-derived antigens can activate dendritic cells (DCs) and trigger anti-tumor immunity [20]. In addition, the tumor-derived DNA can be absorbed by DCs and induce a stronger adaptive anti-tumor immune response.

**Figure 2:**
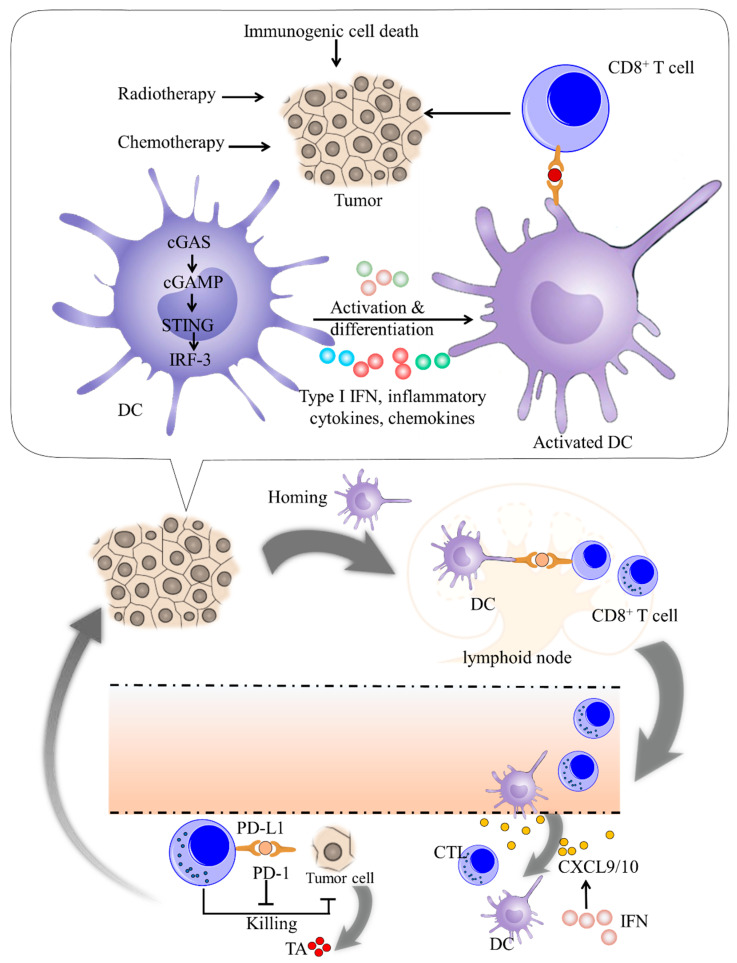
The role of the STING pathway in tumor suppression. In the tumor microenvironment, cGAS-STING in DCs plays an important role in the cross-presentation and priming of tumor-specific CD8+ T cells. Tumor-derived DNA can be taken up by DCs such as protein antigen, resulting in the upregulation of type I IFN. Type I IFN reinforces the cross-presentation of DCs by promoting antigen retention and CD8+ DCs survival. DCs cultured with type I IFN show increased expression of CCR7, which indicates an improved lymph node-homing capability. Additionally, type I IFN upregulates the expression of multiple Th1 chemokines, including CXCL9 and CXCL10, which is important for the homing of APC and trafficking of cytotoxic T lymphocytes. Abbreviations: CTL, cytotoxic T lymphocytes; CXCL9, chemokine (C-X-C motif) ligand 9; IFN, interferon; MHC, major histocompatibility complex; PD-1/PD-L1, anti-programmed death-1/programmed death-ligand 1; TA, tumor antigen; Treg, regulatory T cell.

The cGAS-STING signaling pathway has a critical role in stimulating or enhancing innate and adaptive immunity through cytokines such as type I IFN, promoting the maturation and production of immune cells such as T cells, DCs, and NK cells to trigger effective anti-tumor immune effects [14,21] (Figure 2).

Type I IFN is a cluster of cytokines and is involved in immunomodulation, including 12 IFN-α subtypes, IFN-β, IFN-ε, IFN-κ, and IFN-ω [22]. Type I IFN enhances the production of cytotoxic T cells and improves the specific T cell responses in in vitro experiments [23]. Moreover, type I IFN promotes the stimulation and maturation of DCs, thus facilitating the recruitment of CD4+ T cells and CD8+ T cells [24]. In addition, type I IFN triggers the IFN effect signal pathway of DCs through the paracrine or autocrine, induces the production of MHC I and other co-stimulatory molecules (such as CD86), and promotes DC maturation and cross expression of tumor antigens. Then, mature DCs migrate to lymph nodes, activate CD8+ T cells, and promote anti-tumor cytotoxic T lymphocytes (CTLs) reaction [25,26]. Apart from this, type I IFN can initiate the immune response of NK cells and promote the cytotoxicity of NK cells to tumors [27]. In addition to immune cells, endothelial cells are also important targets of STING agonists. STING agonists can activate the signaling pathway and induce the generation of cytokines such as type I IFN to promote the normalization of the tumor vascular system and tumor microenvironment. Especially when used in combination with antiangiogenic agents, STING agonists can produce more effective anti-tumor outcomes and synergistically improve the efficacy of antiangiogenic agents [28]. Furthermore, the cGAS-STING signaling pathway is reported to be effective in the defense against intracellular DNA and RNA viruses [29,30]. It was demonstrated that cGAS-STING signaling is vital for the host defense against virus infection, as proved by the evidence that fibroblasts and bone marrow-derived macrophages from cGAS^−/−^ mice were defective in IFN-β production and viral clearance during DNA virus MHV68 infection, and cGAS^−/−^ mice were also more susceptible to RNA virus lethal infections [31]. It is possible that DNA from host cells destroyed by viruses may activate the STING pathway for the host defense, and the induction of type I IFNs and ISGs may provide protection against a large variety of microbial infections and improve immune responses [32]. These findings indicate that the STING signaling pathway has a crucial role in anti-tumor immune responses and improving the tumor microenvironment, which can be developed as adjuvants in cancer treatment.

On the contrary, stimulation of the cGAS-STING pathway may result in tumor development and metastasis as well. Investigations have demonstrated that activation of the STING signaling pathway causes indolamine 2,3-dioxygenase (IDO)-dependent effector T cell inhibition, promotes regulatory T cells (Tregs) activity, and induces immune tolerance, leading to tumor growth [33]. It was also elucidated that STING stimulation causes an enhanced increase in brain metastatic cells [34]. Moreover, STING activation could be restrained by gene mutations or gene silencing of STING or cGAS. For instance, KRAS- and LKB1-mutated epigenetic silencing of STING leads to facilitated immune escape in non-small cell lung cancer (NSCLC) cells [35]. Thus, considering the potential opposite function after activating STING, the balance between the cancer immune response and tumor immune evasion, the treatment window, and possible side effects should be considered. The anti-tumor and tumorigenic effects of the STING pathway in different cells and microenvironments need to be evaluated before use as an anti-tumor treatment. When the cGAS-STING signaling pathway is combined with the immune checkpoint blocking therapy (such as PD-L1, PD-1, CTLA-4 inhibitors), it may promote the clinical application of the STING activation strategy.

## 3. Activation of STING Applied to Cancer Immunotherapy

Since the cGAS-STING pathway has a vital role in immunomodulation against tumorigenesis and cancer development, the activation of STING is expected to be used in cancer immunotherapy. Investigations have demonstrated that appropriate strategies can be selected according to the pathological conditions of the tumor to trigger the STING pathway and promote anti-tumor effects. The activation strategies of the cGAS-STING signaling pathway mainly refer to STING agonists, radiotherapy, and chemotherapeutic drugs. 

### 3.1. STING Agonists

The cGAS-STING pathway is particularly important for innate immune sensing of immunogenic tumors. Activation of the cGAS-STING pathway can promote the maturation of antigen-presenting cells (APCs), induce the generation of cytokines and the production of CD8+ T cells against tumor antigens [36], and, at last, reshape the tumor microenvironment and enhance the anti-tumor immune response. Small molecules that activate the cGAS-STING pathway can promote the anticancer effect. STING agonists are mainly divided into 5,6-dimethylxanthenone-4-acetic acid (DMXAA), cyclic dinucleotides (CDNs) and their derivatives, and other small molecule agonists.

#### 3.1.1. DMXAA

DMXAA, also named ASA404, is a STING agonist from the analog of flavone 8-acetic acid, which was originally used in pre-clinical trials as a vascular disrupting agent [37]. DMXAA was found to directly interact with STING and was demonstrated to be effective in potentiating the anti-tumor effect in mice models [38]. A single-arm phase II study investigated the safety and possibility of DMXAA (ASA404) combined with the standard therapy of carboplatin and paclitaxel in patients with advanced NSCLC. The combination was well-tolerated and resulted in no cardiac adverse events or other side effects, which demonstrated improvements in efficacy variables and survival of advanced NSCLC [39]. However, when the scheme was tested in the subsequent phase III clinical trial, no effect was found in the survival and progression-free survival in the DMXAA treatment groups compared with the placebo groups in patients with advanced NSCLC [40]. Unfortunately, human STING cannot be activated by DMXAA. This result is converse to pre-clinical investigation in mice models, in which DMXAA induced significant innate immune responses and potent anti-tumor effects in mice [36,38]. Scientists are still seeking resolutions of DMXAA in the human body. Mechanistically, DMXAA can stimulate the production of NF-κB in endothelial cells [41] and tumor cells [42]. Accumulated NF-κB signals might induce the generation of inflammatory cytokines, modulating immune responses in the tumor microenvironment. The possibility is that DMXAA could promote the translocation of phosphorylated IRF dimers to the nucleus while also stimulating NF-κB targets [43].

#### 3.1.2. CDNs and Derivatives

CDNs, another kind of STING agonist, are a cluster of cyclic dinucleotide family, which can trigger the STING pathway directly. CDNs are derived from bacteria, mainly comprising cyclic di-GMP (c-di-GMP), cyclic di-AMP (c-di-AMP), and cyclic AMP-GMP (cGAMP) molecules. The potential anti-tumor effect was first proved in c-di-GMP, which effectively suppressed both basal and growth factor-induced proliferation in human colon carcinoma H508 cells in vitro [44]. It was subsequently elucidated that CDNs are an essential inducer in activating the host immune response [45,46,47]. For example, intraperitoneal injection of low-dose c-di-GMP resulted in eradication of metastases in a metastatic breast cancer mouse model by enhancing the generation of IL-12 and promoting the immune response of CD8+ T cells. Meanwhile, treatment with high-dose c-di-GMP induced the production of caspase-3 and resulted in cancer cell apoptosis [48]. Moreover, intratumoral administration of c-di-GMP improved the survival rate of glioma-bearing mice and promoted type I IFN signaling and CCL5, CXCL10, and T cell migration in the tumor environment [49]. cGAMP is also a commonly used CDNs, which can stimulate the immune system by activating the cGAS-STING pathway [50,51]. 2′3′-cGAMP is a genuine natural CDN. Intratumoral vaccination of 2′3′-cGAMP in multiple mice models, such as 4T1 murine breast cancer, HSC-2 squamous cell carcinomas, CT26 murine colon cancer, and B16F10 murine melanoma, resulted in a transient increase in macrophages in the tumor and enhanced expression of TNFα and chemokines in the tumor microenvironment [50,52]. Co-injection of cGAMP with PD-1 or CTLA-4 inhibitor exhibited an enhanced anti-tumor effect and increased tumor-infiltrating CD8+ T cell responses in a mouse model of melanoma and an ex vivo model of cultured human melanoma explants [52,53]. Co-administration of 2′,3′-cGAMP, E7GRG (HPV 16 E7 protein), and CpG-C adjuvant in a mouse model of cervical cancer led to an enhanced suppression of tumor growth and metastasis [54]. In addition, treatment of 3′3′-cGAMP in mice with lymphocytic leukemia or myeloma caused significant cancer cell apoptosis and tumor inhibition, indicating the potential of 3′3′-cGAMP in immunomodulation [55]. Furthermore, 3′3′-cGAMP administration resulted in prolonged production of STING in the ER or Golgi apparatus in malignant B cell tumors [55]. Taken together, these results suggest the essential role of STING agonists in anti-tumor immunity and cancer immunotherapy. Agonists of STING such as c-di-GMP have been successfully used as cancer adjuvants, and cGAMP was proved to be effective in enhancing the anti-tumor effect when combined with radiotherapy and immune checkpoint inhibitors (ICIs) [53,56,57].

Apart from classic CDNs, derivative CDNs have emerged with an improved performance, which mainly include ADU-S100, MK-1454, SB-11285, ADU-V19, IACS-8779, IACS-8803, and IMSA101.

ADU-S100 (MIW815) was the first CDN agent to be applied in clinical trials of cancer immunotherapy, with enhanced stability and lipophilicity. It was demonstrated that treatment with ADU-S100 resulted in a significant inhibition of tumor growth in mice and led to improved systemic immune responses to decrease far-away metastasis and prolong the immunologic memory in B16 melanoma, CT26 colon cancer, and 4T1 breast cancer models [36]. ADU-S100 combined with PD-L1 modulator and OX40 receptor is effective in activating the innate immunity and conquering the immune tolerance of antigen, in which intratumoral injection of ADU-S100 effectively activates tumor antigen-specific CD8+ T cell responses [58]. Moreover, ADU-S100 could potently prime the production of type I IFN, thus reducing abnormal tumor vasculature formation and promoting the expression of CD8+ T cells, and thereby inhibiting the tumor formation. In addition, ADU-S100/MIW815 was investigated in clinical studies. In a phase I clinical trial (NCT02675439), the safety of intratumoral administration of ADU-S100 was assessed in 40 patients with advanced/metastatic solid tumors or lymphomas, and no toxicity to hosts were reported [59]. Treatment of ADU-S100 plus the PD-1 inhibitor spartalizumab was investigated in a phase Ib clinical trial (NCT03172936) to characterize the safety and scientific validity of the combination in patients diagnosed with advanced/metastatic solid tumors or lymphomas [60]. Moreover, co-injection of ADU-S100 and pembrolizumab was studied in patients with PD-L1-positive recurrent or metastatic HNSCC (NCT03937141), and the combination of ADU-S100 with ipilimumab was tested clinically in patients with advanced/metastatic solid tumors or lymphomas (NCT02675439).

MK-1454 is a synthetic CDN derivative with an advanced performance. The prior phase I study (NCT03010176) on MK-1454 demonstrated good activity and safety in patients with advanced solid tumors or lymphomas when injected with MK-1454 alone or in combination with ICI pembrolizumab [61]. Moreover, intratumoral administration of MK-1454 individually or combined with pembrolizumab was assessed in patients with metastatic or unresectable recurrent HNSCC (NCT04220866).

SB-11285 is a CDN derivative STING agonist. It was proved in a pre-clinical study that intratumoral treatment of SB11285 leads to sufficient suppression of tumor growth in mice. Moreover, co-administration of SB11285 with cyclophosphamide had a synergistic anti-tumor effect and promoted the immune responses [62]. Furthermore, intravenous injection of SB11285 alone or in combination with Atezolizumab was evaluated in a phase 1a/1b study patients with advanced solid tumors (NCT04096638). This investigation aimed to prove the anti-tumor activity of intravenous SB11285 as an adjuvant and determine the dose-limiting toxicities and tolerated dose of SB11285.

ADU-V19 is a modified human STING agonist. As a CDN analog, ADU-V19 has similar efficacies to those of CDN STING agonists, which can activate type I IFN and induce T cell responses [63].

IACS-8779 and IACS-8803 are derived from CDN STING agonists that are highly efficient in activating the STING pathway in vitro. Intratumoral injection of IACS-8779 or IACS-8803 in mice with B16 melanoma tumors led to a robust systemic anti-tumor efficacy [64].

IMSA101 is a cGAMP analog whose structure has not been reported. In order to investigate the safety and clinical efficacy of IMSA101, it was evaluated individually and combined with ICI in phase I/IIa studies (NCT04020185) of patients with solid or refractory malignancies.

#### 3.1.3. Non-CDN Agonists

Other small-molecule agonists mainly refer to non-CDNs. E7766 pertains to the kind of STING agonists with a macrocycle bridge, which may match both human and mouse STING protein. It was proved that administration of E7766 (i.t.) in mice led to a significant reduction in subcutaneous tumor growth [65]. Treatment with E7766 (i.v.) demonstrated enhanced IFN-β gene induction and tumor inhibition in a dose-dependent manner in mice with BCG-unresponsive non-muscle invasive bladder cancer [66]. Moreover, injection of E7766 led to 90% tumor regression in mice bearing CT26 tumors with no recrudescence for 8 months [67]. In addition, the clinical efficacy of E7766 was also assessed in a phase 1/1b clinical trial, in which treatment with E7766 (i.t.) is regarded as a monotherapy in patients with advanced solid tumors or lymphomas (NCT04144140) and in patients diagnosed with non-muscle invasive bladder cancer (NCT04109092). MK-2118 is a STING agonist with an unknown structure. In order to evaluate the safety, tolerability, and MTD of MK-2118, its was tested in patients with advanced solid tumors or lymphomas (NCT03249792) and administrated intratumorally or subcutaneously, alone or in combination with pembrolizumab. Moreover, amidobenzimidazole (ABZI)-based analogs were designed to improve systemic delivery, which can bind to the C-terminal domain of STING and enhance the biding affinity. The representative one is diABZI from linked ABZIs, which showed a potent effect in ameliorating the affinity to STING and inducing the secretion of IFN-β in human PBMCs. Administration of diABZI in mice bearing CT26 colorectal tumors resulted in significant tumor inhibition and enhanced survival, with 80% of mice being tumor free [68]. Compound 3, an ABZI-based compound, can trigger STING and produce IFN-β, suppress CT26 colorectal tumor growth in mice, and improve the survival rate by enhancing T cell immune responses [68]. MSA-2, an orally available small-molecule STING agonist, was demonstrated to be effective in inducing tumor regression in mice with durable anti-tumor immunity and activating IFN-β secretion in various syngeneic murine tumor models [69]. In recent studies, JNJ-‘6196 has been projected to be the next-generation STING agonist, which can trigger DCs and induce the production of cytokines with high potency. Treatment with JNJ- ‘6196 in mice resulted in tumor regression, improved immune resistance, and enhanced ICI effects in PD-1 non-responsive tumor models [70]. The efficacy of JNJ-‘6196 in synergistically improving the effects of ICI makes it a potent adjuvant for clinical treatments.

#### 3.1.4. Bacterial Vectors

Apart from the above STING agonists, bacterial vectors are considered as novel delivery approaches for STING agonists to transform into specific cells. One vector is SYNB1891, which is localized to the tumor microenvironment and expresses the enzymes to generated c-di-AMP. Intratumoral injection of SYNB1891 in mice bearing B16.F10 melanoma tumors resulted in the generation of type I IFNs and tumor regression [71]. Moreover, the anti-tumor efficacy of intratumoral SYNB1891 is also being tested in an ongoing phase I clinical trial of patients diagnosed with advanced/metastatic solid tumors and lymphoma (NCT04167137). Another bacterial vector is STACT, which carries an inhibitory microRNA to TREX-1 (inhibiting the STING pathway). Scientific studies have demonstrated that treatment with STACT-TREX-1 resulted in specific colonization of the myeloid [72] and tumor regression and durable immunity in CT26 and MC38 murine models [73].

#### 3.1.5. STING Agonists in Clinical Trials

The cGAS-STING pathway can activate type I IFN and have crucial roles in anti-tumor immunity. STING agonists targeting the signaling pathway have demonstrated great potential in improving the tumor environment, enhancing the anti-tumor immune response, and promoting anti-tumor effects. Thus, the STING agonists that have been applied in important clinical trials are summarized in Table 1 [74,75,76,77,78,79,80,81,82,83,84,85,86], administrated alone or in combination with other cancer therapies. The clinical trials are indicated on the website https://ClinicalTrials.gov (accessed on 18 June 2022).

### 3.2. Radiotherapy

Radiotherapy usually causes the destruction of nuclear and gene instability in cancer cells [87], and the accumulated tumor pathological DNA in the cytoplasm may activate the cGAS-STING signal pathway, and then induce congenital immunity and promote the adaptive immune response. Recent studies have proved that radiotherapy treatment and the resulting anti-tumor efficacy is closely related to the activation of the cGAS-STING pathway and production of type I IFN. Treatment with cGAMP could greatly improve the activation of the cGAS-STING pathway. Deng et al. illustrated that mice with a STING or cGAS deficiency were not activated by a valid dose of radiation, and DCs could not be induced by type I IFN, even after radiotherapy treatment. They also proved that the cGAS-STING-dependent cytoplasmic DNA sensing pathway is necessary for a radiation-activated anti-tumor adaptive immune response [88]. However, whether radiotherapy induces cGAS-STING-mediated anti-tumor effects or not is related to the applied radiation dose. The dose of radiation is vital for STING pathway activation. It has been demonstrated that low-dose radiation may trigger insufficient biological responses while overdosed radiation may cause adverse effects [89,90]. In addition, Luo et al. demonstrated that the enhancement of the T cell response by radiotherapy and nanovaccine is STING dependent. In STING-mutant or -deficient mice, the anti-tumor effect of this strategy is greatly reduced [91]. In a mouse model of homologous melanoma or neuroblastoma, the nanovaccine combined with radiotherapy also activated DCs and effector T cells, resulting in obvious tumor regression and specific anti-tumor immune memory [92]. A kind of liposome nanoparticle loaded with cGAMP in combination with radiotherapy triggered the generation of type I IFN in APC and resulted in specific anti-tumor feedback in a mouse model of lung metastasis [93].

### 3.3. Chemotherapeutic Drugs

Many anti-tumor chemotherapeutic drugs are cytotoxic and may destroy chromosomal DNA. When damaged DNA fragments accumulate in the cytoplasm and are recognized by cGAS, a type I IFN reaction is triggered to produce immune regulation. The chemotherapies etoposide and cisplatin can induce intrinsic STING-dependent cytokine production through DNA damage. It was demonstrated that etoposide activated the STING pathway via the production of DNA adducts of ssDNA and dsDNA, and cisplatin can interfere with DNA repair, cause DNA damage, and trigger the STING pathway [94]. Grabosch et al. evaluated the tumor immunogenicity and in vivo anti-tumor effect of cisplatin in two new mouse models of ovarian cancer, when used as monotherapy or combination therapy with PD-L1 inhibitors. It was found that acute and chronic exposure to cisplatin leads to the accumulation of T cells in tumors and causes an increase in the expression level of calreticulin, MHC I, and other molecules during the process of antigen presentation [95]. In addition, it was also demonstrated that long-term exposure to DNA-damaging substances leads to nucleosome leakage and triggers STING-dependent cytoplasmic DNA signal transduction [96]. Moreover, Li et al. proved that co-administration of cGAMP and 5-FU can significantly suppress tumor growth in mice, improve the survival rate, and significantly alleviate the toxicity and side effects of intestinal epithelial tissue damage and mucosal atrophy caused by 5-FU in a mouse colon cancer model [51].

## 4. Combination of STING Agonists with Other Cancer Therapies

The activation of the cGAS-STING pathway may induce various cytokines and stimulate adaptive immunity. When STING agonists are combined with other cancer therapies, it might achieve positive feedback, improve the tumor microenvironment, and lead to a sustained anti-tumor effect [97].

### 4.1. STING Agonists and Chemotherapies

Chemotherapy, as a cancer therapy, may induce damaged ssDNA or dsDNA accumulation, which could trigger the signaling pathway of cGAS-STING and enhance T cell response [98]. It was elucidated that administration of phosphatidylserine loaded with cGAMP-adenosine monophosphate (NP-cGAMP) resulted in rapid spread of NP-cGAMP to specific sites, activation of the cGAS-STING pathway and production of type I IFN, and inhibition of lung metastasis in mouse models [83]. Moreover, when 5-FU was combined with cGAMP, the combination demonstrated improved innate immune responses, enhanced anti-tumor efficacies, and reduced the side effects of 5-FU as well [44]. Furthermore, the anthracycline antibiotics, such as adriamycin, can activate the production of IFN and trigger the cGAS-STING pathway [99].

### 4.2. STING Agonists and Radiotherapy

Radiation-induced DNA damage can trigger the cGAS-STING pathway and induce T cell responses [88], and the combination of radiotherapy with STING agonists produces a better synergistic anti-tumor effect. Xue et al. found that the STING agonist diABZI enhanced the radiosensitivity of NSCLC cells to irradiation via stimulation of the cGAS-STING pathway and promotion of apoptosis [100]. Moreover, it was demonstrated in two mouse models that the combination of local radiation and PC7A nanovaccine could synergistically activate the STING signal transduction pathway and lead to a better therapeutic anti-tumor efficacy, which significantly improved the immunosuppression and survival time in tumor-bearing mice. In addition, the enhancement of the T cell response by radiotherapy and nanovaccine is STING dependent, and this strategy could not work or was reduced in STING-mutant or -deficient mice [91]. In a mouse model of homologous melanoma or neuroblastoma, a nanovaccine with a PC7A/CPG composite nucleus combined with radiotherapy activated DCs and effector T cells, inducing obvious tumor inhibition and specific anti-tumor immune memory [92]. A kind of liposome nanoparticle loaded with cGAMP in combination with radiotherapy could inhibit lung metastasis of 4T1 breast cancer [93]. Jagodinsky et al. demonstrated that targeted radionuclide therapies (TRTs) can potently stimulate type I IFN, which is vital for the combination of TRT with immunotherapies [101].

### 4.3. STING Agonists and Immunotherapy

STING agonists can activate the cGAS-STING pathway, induce the expression of type I IFN, improve the tumor environment, and lead to promoted anti-tumor effects [102]. These characteristics make STING agonists an ideal candidate for combination with ICI (such as anti-PD-1/PD-L1, CTLA-4 antibodies) [103,104], which only work in a fraction of patients with good anti-tumor effects.

It was demonstrated that treatment with cGAMP (i.m.) at a site far away from the tumor could significantly promote the therapeutic effect of immune-checkpoint-blocking. cGAMP, which, combined with PD-L1 antibody, effectively suppressed tumor growth in mice bearing a B16 melanoma tumor [105] while STING-deficient mice showed a weak response to immune-blocking. Ager et al. proved that STING agonist combined with PD-1 inhibitors showed an enhanced anti-tumor effect, especially in tumor models with a poor response to ICI [106]. When nanoparticles loaded with CDNs and PD-1 inhibitor were treated in established B16 melanoma tumors in vivo, the integrated nanoparticles presented a log-fold improvement in the anti-tumor potency compared with free CDN without nanoparticles [107]. Moreover, a vaccine PancVAX-targeted neoantigen, combined with the STING agonist ADU-V16, resulted in stimulation of the T cell repertoire and led to temporary tumor rejection in mice burdened with pancreatic (Panc02) tumors.

When PancVAX was co-administrated with two ICIs (anti-PD-1 and agonist OX40 antibodies), it promoted tumor inhibition and prolonged the survival benefit [63]. Co-treatment of STING agonists with CTLA4 and PD-1 antibodies resulted in a significant survival advantage in a preclinical HPV + oral tumor model when compared with no treatment or treatment with ICI only [108]. In addition, Nakamura et al., found that treatment with STING-LNP (a lipid nanoparticle containing STING agonist) could reverse PD-1 resistance in a mouse model with B16-F10 lung metastasis. Mechanistically, NK cells can be stimulated by the STING pathway, which, in turn, promotes the production of PD-L1 in cancer cells [109]. Apart from that, CAR T cells generated from Th/Tc17 cells together with the STING agonists DMXAA or cGAMP significantly improve tumor control and prolong CAR T cell persistence in the tumor microenvironment in breast cancer [110]. Furthermore, cGAMP was evaluated to be effective in activating NK cells and enhancing the sensitivity of pancreatic cancer cells to NK cells. The integration of CAR-NK-92 cells targeting mesothelin and cGAMP presented better anti-tumor effects compared with individual treatment in a mouse model of pancreatic cancer by improving tumor control and promoting survival [111]. Co-administration of c-di-GMP and a Listeria monocytogenes-based vaccine was also demonstrated to be effective in decreasing the number of MDSCs in blood and inhibiting tumor growth and metastases, indicating the potent prospect of c-di-GMP application as an adjuvant against cancer [48].

STING agonists combined with other drugs have good application prospects, and some combined therapies have entered clinical trials. The efficacy of the STING agonist ADU-S100 combined with the PD-1 antibody pembrolizumab has been clinically tested in head and neck cancer (NCT03937141); ADU-S100 integrated with ipilimumab was assessed in patients with advanced/metastatic solid tumors or lymphomas (NCT02675439). Moreover, co-administration of ADU-S100 and the PD-1 checkpoint inhibitor PDR001 has also been used in patients with advanced/metastatic solid tumors or lymphomas. In addition, another clinical trial of intratumoral injection of MK-1454 combined with intravenous injection of pembrolizumab is under way, as assessed in patients with metastatic or unresectable recurrent HNSCC.

## 5. Conclusions and Future Directions

The cGAS-STING signaling pathway is an important process in cytoplasmic DNA sensing, and has critical roles in regulating pathogen infection, tumor immunity, and autoimmune diseases. Activation of the cGAS-STING pathway can induce the expression of type I IFNs and other cytokines, and conduct the signals to the nucleus, which makes the cGAS-STING pathway a promising target for cancer immunotherapy. Effective STING agonists can improve the tumor microenvironment immunity and enhance the anti-tumor effect. Recently, STING agonists have been investigated in pre-clinical scientific studies and clinical trials. It has been demonstrated that STING agonists have promising biological activity and show excellent synergistic anti-tumor effects in combination with other cancer therapies such as radiotherapy, chemotherapy, or immune therapies, as proven in preclinical studies and some clinical trials. The combination of STING agonists with ICIs (PD-1 inhibitor or CTLA-4 antibody) has presented significant synergistic anti-tumor effects in several tumor models, and the clinical efficacy of STING agonists integrated with PD-1 antibody is under investigation in patients for cancer immunotherapy.

Even though STING agonists have demonstrated good application prospects, the clinical application of the cGAS-STING signal pathway in cancer treatment is limited at present. Given the complexity of the immune network and safety problems, attention should be given to the treatment window, toxicity, and side effects of STING-activation-based combination therapy to promote therapeutic efficacies and avoid excessive activation of the STING pathway. In summary, the cGAS-STING pathway has great potential in cancer immunotherapy as it improves the immune ability and facilitates the combined cancer biotherapeutic efficacies. In-depth research on the clinical application of STING agonists will not only deepen the understanding of innate and adaptive immunity and help to develop more effective STING agonists as anti-tumor drugs but also provide a theoretical basis for the anti-tumor immunotherapy strategy and its combined applications.

## Figures and Tables

**Table 1:** Summary of STING agonists used in clinical trials.

Drug	Administration	Phase	Cancer Type	Clinical Trial NCT Number	Patients	References
**ASA404 + Paclitaxel + carboplatin**	i.v.	I/II	Advanced and metastatic NSCLC	NCT00832494	105	[74]
**ASA404 + Paclitaxel + carboplatin**	i.v.	IIIb/IV	NSCLC	NCT00662597	1285	[75]
**ADU-S100 +/− ipilimumab**	ADU-S100(i.t.) ipilimumab(i.v.)	I	Advanced/Metastatic solid tumors or lymphomas	NCT02675439	47	[76]
**ADU-S100 +/− PDR001**	ADU-S100(i.t.) PDR001(i.v.)	Ib	Advanced/Metastatic solid tumors or lymphomas	Nct03172936	106	[77]
**ADU-S100 +/− perbrolizumab**	ADU-S100(i.t.) perbrolizumab(i.v.)	II	Recurrent and metastatic HNSCC advanced solid tumors	NCT03937141	33	[78]
**MK-1454 +/− perbrolizumab**	MK-1454(i.t.) perbrolizumab(i.v.)	I	Advanced/Metastatic solid tumors or lymphomas	NCT03010176	235	[79]
**MK-1454 +/− perbrolizumab**	MK-1454(i.t.) perbrolizumab(i.v.)	II	Metastatic or unresectable, recurrent HNSCC	NCT04220866	200	[80]
**SB 11285 + Atezolizumab**	SB 11285(i.v.) Atezolizumab	Ia/Ib	Melanoma, HNSCC and advanced solid tumor	NCT04096638	110	[81]
**IMSA101 +/− ICI**	IMSA101(i.t.) ICI	I/IIa	Advanced treatment- refractory malignancies	NCT04020185	115	[82]
**E7766**	i.t.	I/Ib	Advanced solid tumors or lymphomas	NCT04144140	120	[83]
**E7766**	i.t.	I/Ib	Non-muscle invasive bladder cancer	NCT04109092	120	[84]
**MK-2118 +/− perbrolizumab**	i.t.	I	Advanced/Metastatic solid tumors or lymphomas	NCT03249792	160	[85]
**SYNB1891 +/− Atezolizumab**	i.t.	I	Advanced solid tumors or lymphomas	NCT04167137	70	[86]

Abbreviations: NSCLC: Non-small cell lung cancer; HNSCC: head and neck squamous cell carcinoma; i.t.: intratumoral injection; i.v. intravenous injection; ICI: immune checkpoint inhibitor; +/−: combination/alone.
